# Supplementary material for: Glioma-initiating cells at tumor edge gain signals from tumor core cells to promote their malignancy
Source: Nat Commun. 2020 Sep 16;11:4660. doi: 10.1038/s41467-020-18189-y (PMC7494913; doi:10.1038/s41467-020-18189-y)
Supplement: Supplementary file 1 — Supplementary information [file 41467_2020_18189_MOESM1_ESM.pdf]

Supplementary information

Glioma-initiating cells at tumor edge gain signals from tumor core cells to promote their malignancy

Soniya Bastola, Marat S. Pavlyukov, Daisuke Yamashita, Sadashib Ghosh, Heejin Cho, Noritaka Kagaya, Zhuo Zhang, Mutsuko Minata, Yeri Lee, Hirokazu Sadahiro, Shinobu Yamaguchi, Svetlana Komarova, Eddy Yang, James Markert, Louis B. Nabors, Krishna Bhat, James Lee, Qin Chen, David K. Crossman, Kazuo Shin-Ya, Do-Hyun Nam, and Ichiro Nakano

Supplementary Figures 1 - 9

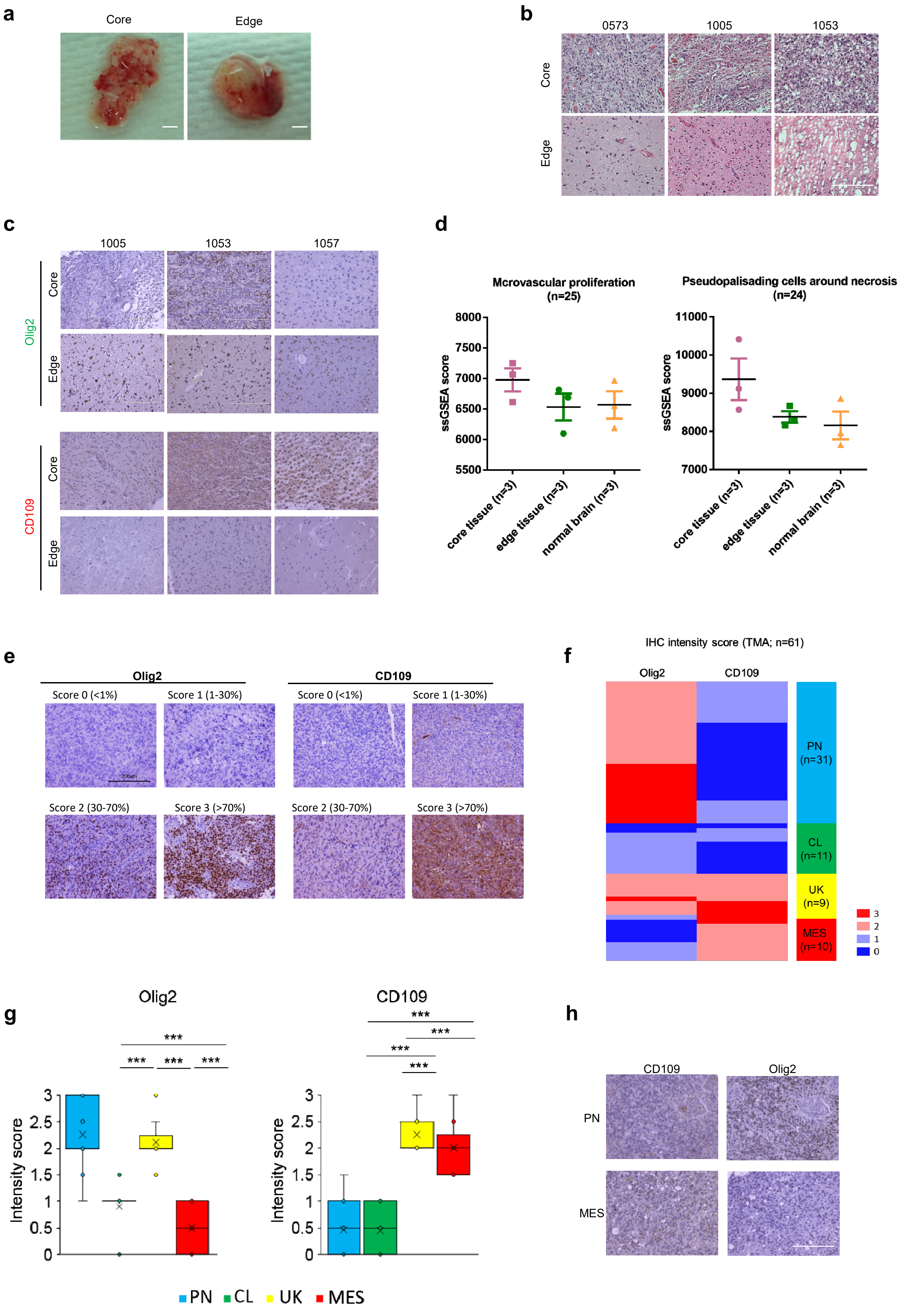

a
Core
Edge
b
0573
1005
1053
Core
Edge
c
1005
1053
1057
Olig2
Core
Edge
CD109
Core
Edge
d
Mcrovascular proliferation (n=25)
Pseudopalisading cells around necrosis (n=24)
ssGSEA score
8000
7500
7000
6500
6000
5500
11000
10000
9000
8000
7000
core tissue (n=3)
edge tissue (n=3)
normal brain (n=3)
e
Olig2
CD109
Score 0 (<1%)
Score 1 (1-30%)
Score 2 (30-70%)
Score 3 (>70%)
f
IHC intensity score (TMA; n=61)
Olig2
CD109
PN (n=31)
CL (n=11)
UK (n=9)
MES (n=10)
3
2
1
0
g
Olig2
CD109
***
Intensity score
3
2.5
2
1.5
1
0.5
0
PN
CL
UK
MES
h
CD109
Olig2
PN
MES

Supplementary Figure 1 GBM cells at the invading edge exhibit distinct molecular signature from those localized in the core. **a** Representative surgical specimens of core and edge tissues from patient #101027. Scale bar 1 mm. **b** H&E staining of edge (lower) and core (upper) tumor tissue from three GBM patients. Scale bar 200 µm. **c** IHC staining of paired edge and core tumor tissues for Olig2 (upper) and CD109 (lower). Scale bar 200 µm. **d** Single sample gene set enrichment analysis (ssGSEA) of normal brain, edge and core tumor tissues using microvascular proliferation (MVP) and pseudo-palisading cells around necrosis (PAN) gene signatures from Ivy Glioblastoma Atlas Project database. n = 3 independent samples per group. Data are mean ± s.d. **e** Representative IHC staining of human GBM tissues for Olig2 (left) and CD109 (right) illustrating different staining intensities. Scale bar 200 µm. **f** Heatmap showing the staining intensity of Olig2 and CD109 in all three subtypes of GBM, n = 61. **g** Quantification of IHC staining intensity for Olig2 and CD109 of GBM samples obtained from 61 patients and related to proneural (n = 31 independent samples), classical (n = 11 independent samples), unknown (n = 9 independent samples) and mesenchymal (n = 10 independent samples) subtypes. The line in the box is the median, the left and right of the box are the first and third quartiles, and the whiskers extend to 10th and 90th percentiles respectively. ***$p<0.001$ using one-way ANOVA followed by Tukey's posttest. **h** IHC staining of PN and MES GBM tumor tissues for Olig2 and CD109. Scale bar 200 µm.

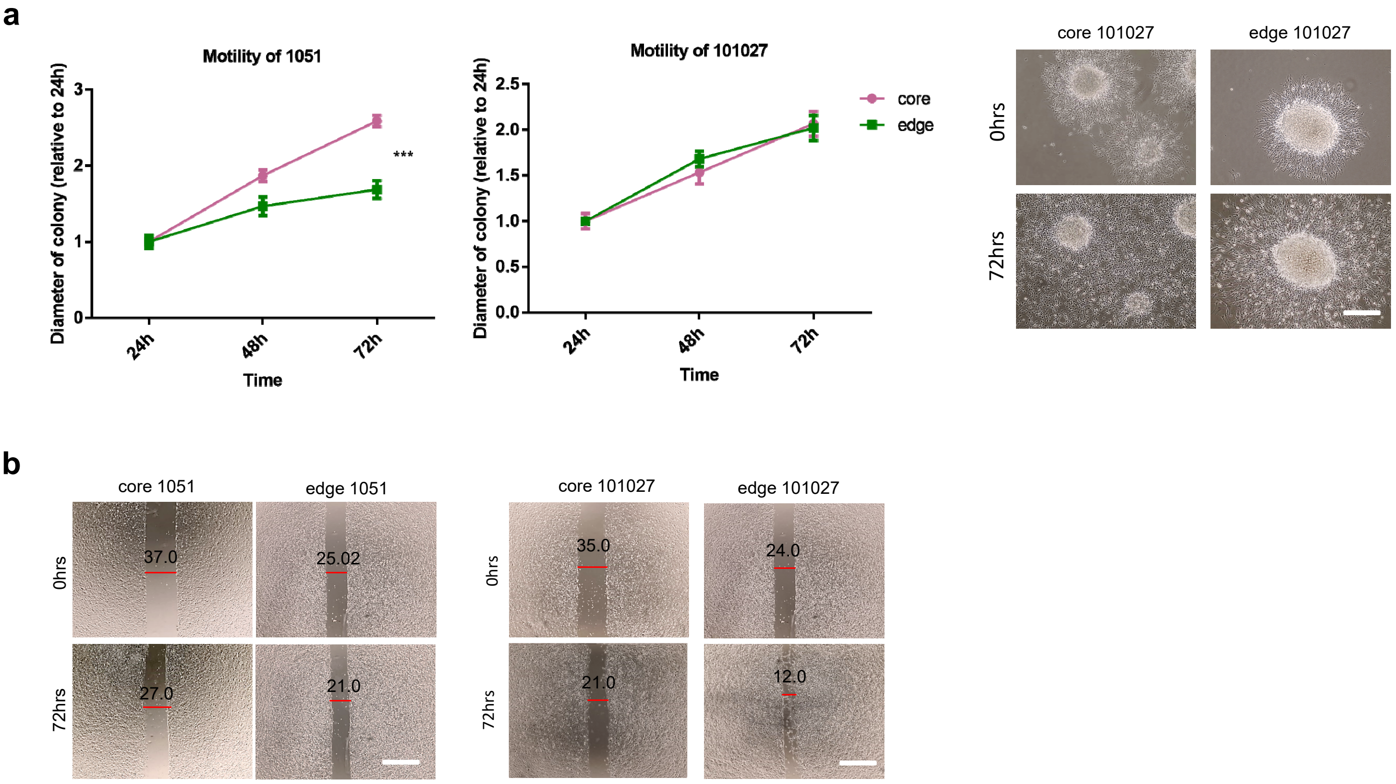
a
Motility of 1051
Diameter of colony (relative to 24h)
***
24h
48h
72h
Time
Motility of 101027
core
edge
core 101027
edge 101027
0hrs
72hrs
b
core 1051
edge 1051
core 101027
edge 101027
37.0
25.02
27.0
21.0
35.0
24.0
21.0
12.0

Supplementary Figure 2 *In vitro* migration of regionally-specified GBM cells. **a** *In vitro* cell motility assay comparing 1051 and 101027 edge and core sphere colony motility at 24, 48 and 72 hours (left), with representative images (right). Scale bar 100 um. ***p<0.001 using unpaired, two-tailed t test. Data are mean ± s.d; n = 3 independent samples per group. **b** Wound healing assay comparing core (left) and edge (right) 1051 sphere migration after 72 hours. Scale bar 200 um.

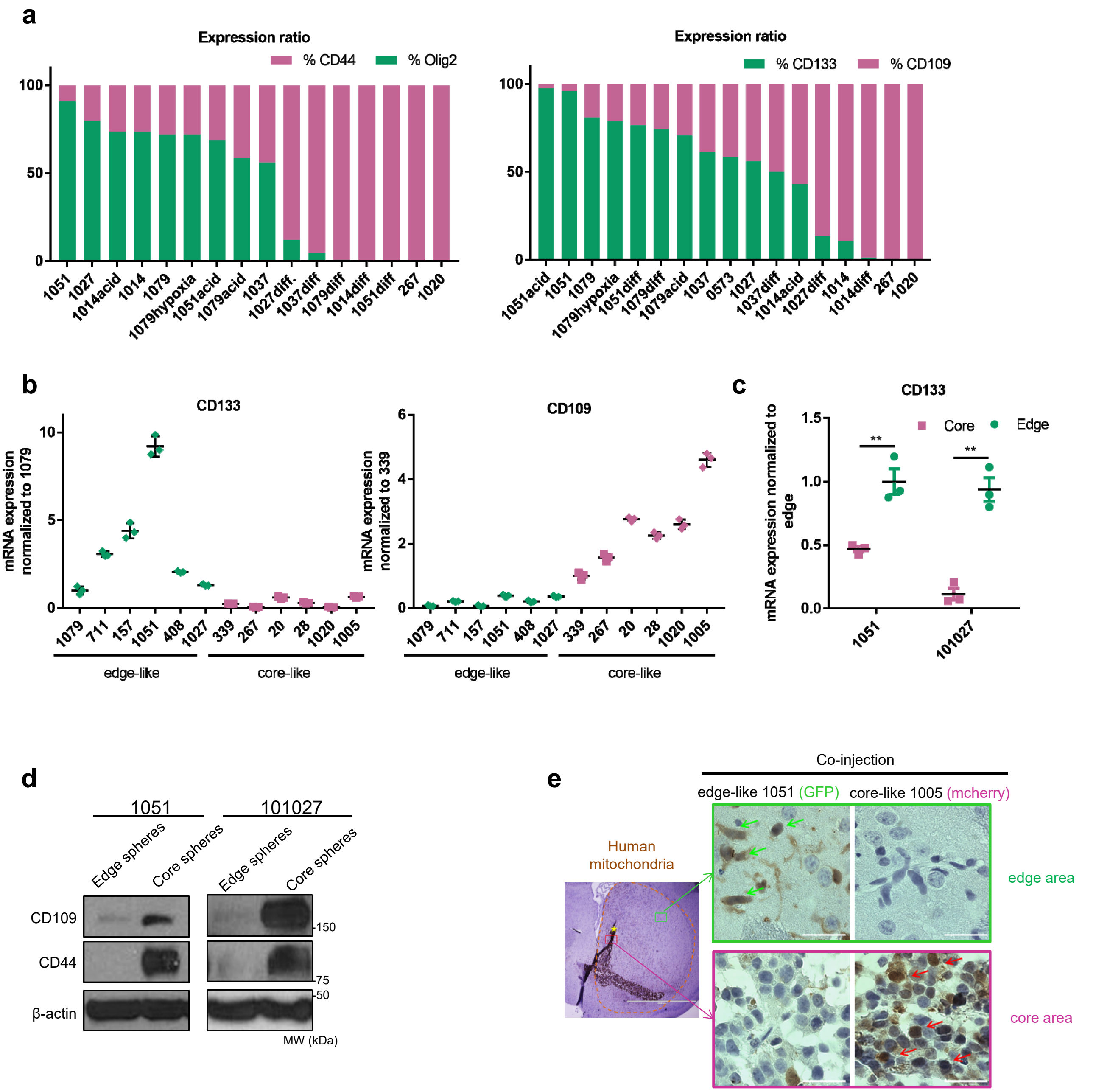

f

Characteristic of tumor tissues and GBM spheres

		Transcriptional signature		Malignant biological behavior		
		markers	pathways	Infiltration	location preference	IR-resistance
Tissue	edge	Olig2	KRAS	-	-	-
	core	CD44/CD109	c-MYC/G2M check point	-	-	-
Spheres	edge-like	CD133	KRAS	high	edge	low
	edge	CD133	KRAS	high	edge	low
	core-like	CD109	c-MYC/G2M check point	low	core	high
	core	CD109	c-MYC/G2M check point	low	core	high

Supplementary Figure 3 Edge-like and core-like GBM sphere lines retain key properties of spatially distinct edge and core GBM. **a** Expression ratio of CD44/Olig2 (left) and CD133/CD109 (right) in various GBM spheres calculated from RNA-seq data. **b** qRT-PCR analysis of CD133 and CD109 expression in six edge-like and six core-like GBM sphere lines. Data are mean ± s.d.; n = 3 independent samples per group. **c** qRT-PCR analysis of CD133 expression in two edge and two core sphere lines. Data are mean ± s.d. **$p<0.01$ using unpaired, two-tailed t test.; n = 3 independent samples per group. **d** WB for CD109 and CD44 using paired 1051 and 101027 edge and core GBM sphere lines. **e** IHC staining for human mitochondria (left), GFP (middle) and mCherry (right) of mouse brain co-injected with edge-like 1051 (GFP labeled) and core-like 1005 (mCherry labeled) GBM spheres at 1:1 ratio. Different regions of the tumor are magnified: edge (upper) and core (lower). Scale bar 2 mm (left) and 20 um (right). **f** Table showing the similarity between edge-like/core-like spheres, regionally-defined edge/core spheres and edge/core GBM tumor tissues.

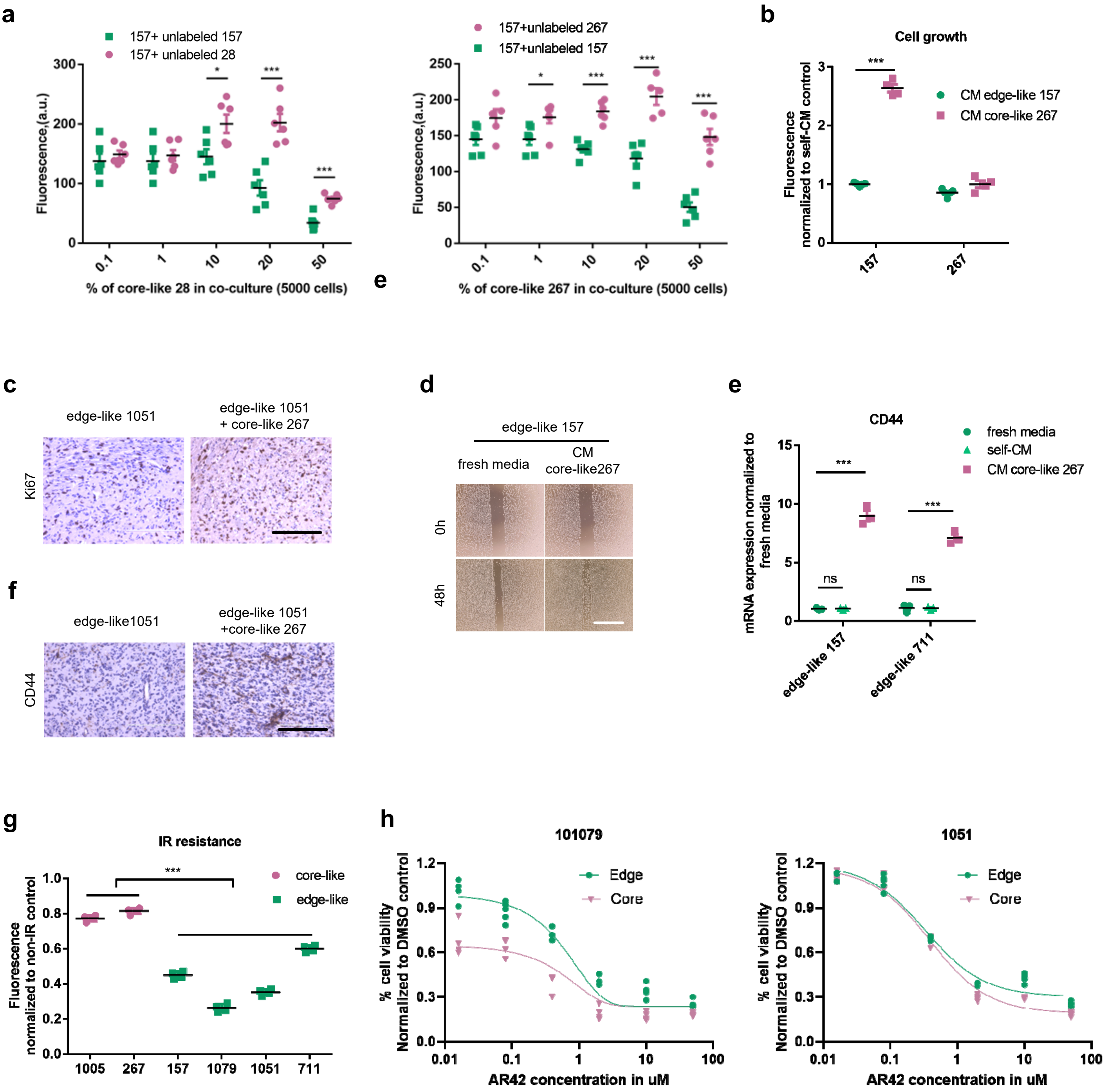
a
157+ unlabeled 157
157+ unlabeled 28
Fluorescence,(a.u.)
% of core-like 28 in co-culture (5000 cells)
157+unlabeled 267
157+unlabeled 157
% of core-like 267 in co-culture (5000 cells)
e
b
Cell growth
CM edge-like 157
CM core-like 267
Fluorescence normalized to self-CM control
c
edge-like 1051
edge-like 1051 + core-like 267
Ki67
d
edge-like 157
fresh media
CM core-like267
0h
48h
e
CD44
fresh media
self-CM
CM core-like 267
mRNA expression normalized to fresh media
edge-like 157
edge-like 711
f
edge-like1051
edge-like 1051 +core-like 267
CD44
g
IR resistance
core-like
edge-like
Fluorescence normalized to non-IR control
1005
267
157
1079
1051
711
h
101079
1051
Edge
Core
% cell viability Normalized to DMSO control
AR42 concentration in uM

Supplementary Figure 4 Intercellular signaling from core GBM cells provokes aggressiveness of the edge counterparts. **a** Fluorescence intensity of mCharry labeled edge-like 157 GBM spheres co-cultured with increasing amounts of unlabeled edge-like 157 GBM spheres, unlabeled core-like 28 (left) or unlabeled core-like 267 (right) GBM spheres. *$p<0.05$, ***$p<0.001$ using unpaired, two-tailed t test. Data are mean ± s.d.; n = 6 independent samples per group. **b** *In vitro* cell growth assay of edge-like 157 and core-like 267 GBM cells treated with CM from edge-like 157 or core-like 267 GBM spheres. ***$p<0.001$ using unpaired, two-tailed t test. Data are mean ± s.d.; n = 5 independent samples per group. **c** IHC staining for Ki67 of mouse brain intracranially injected with edge-like 1051 GBM spheres alone or with core-like 267 GBM spheres (ratio 95:5). Scale bar 200 μm. **d** Wound healing assay of edge-like 157 GBM spheres treated with/without CM from core-like 267 GBM spheres. Scale bar 100 um. **e** qRT-PCR analysis of CD44 expression using edge-like 157/711 GBM spheres treated with/without CM from core-like 267 GBM spheres. ***$p<0.001$ using one-way ANOVA followed by Dunnett's posttest. Data are mean ± s.d.; n = 3 independent samples per group. **f** IHC staining for CD44 of mouse brain intracranially injected with edge-like 1051 GBM spheres alone or with core-like 267 GBM spheres (ratio 95:5). Scale bars 200 μm. **g** *In vitro* cell viability assay of edge-like (157, 1079, 1051, 711) and core-like (1005, 267) GBM spheres irradiated with 4Gy (IR). ***$p<0.001$ using unpaired, two-tailed t test. Data are mean ± s.d.; n = 5 independent samples per group. **h** *In vitro* cell viability assay of 101079 and 1051 edge or core GBM spheres treated with DMSO or AR42 at different concentrations. Data are mean ± s.d.; n = 3 independent samples per group.

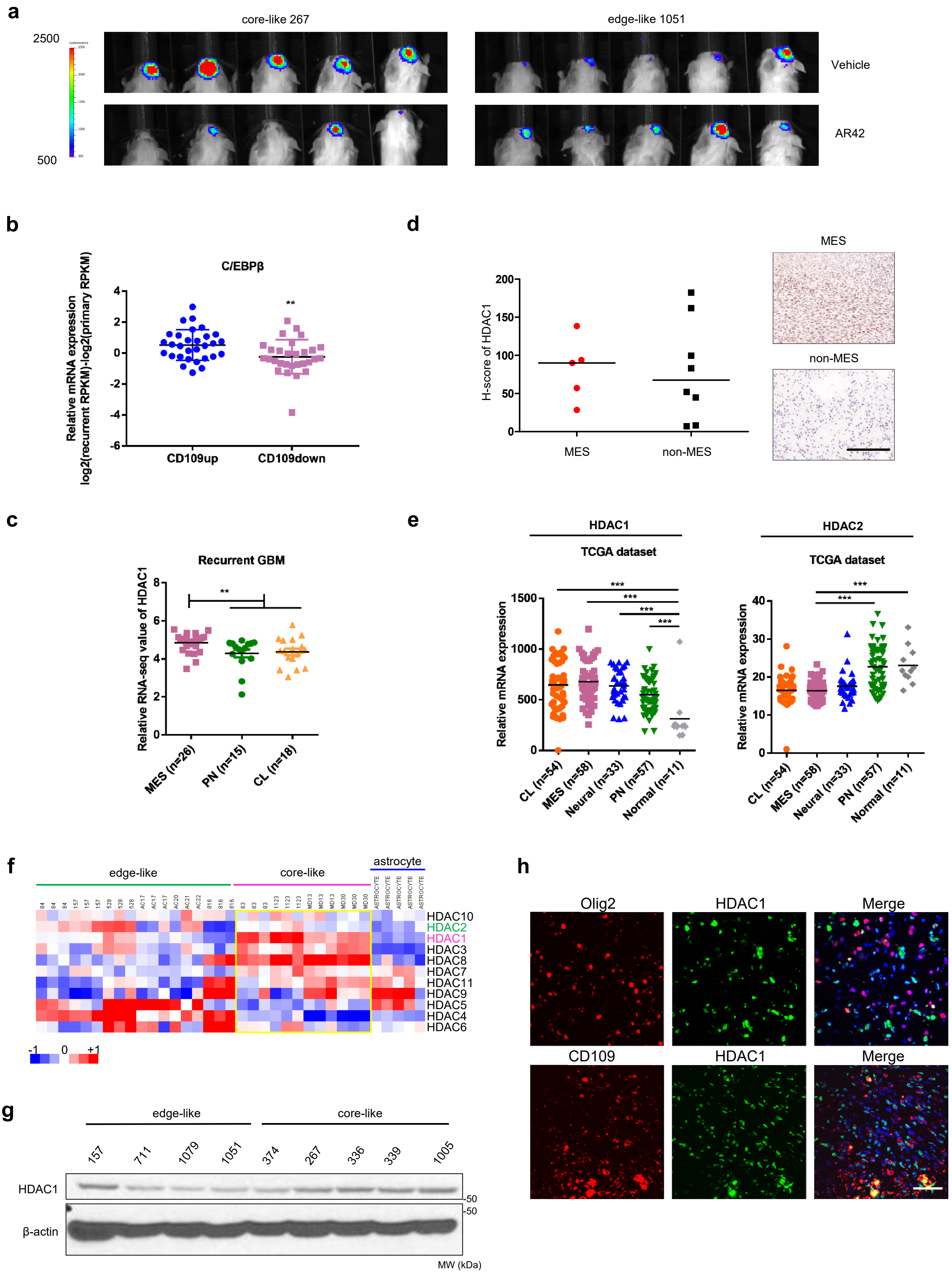

a
core-like 267
edge-like 1051
2500
500
Vehicle
AR42
b
C/EBPβ
Relative mRNA expression
log2(recurrent RPKM)-log2(primary RPKM)
CD109up
CD109down
d
MES
non-MES
H-score of HDAC1
c
Recurrent GBM
Relative RNA-seq value of HDAC1
MES (n=26)
PN (n=15)
CL (n=18)
e
HDAC1
HDAC2
TCGA dataset
Relative mRNA expression
CL (n=54)
MES (n=58)
Neural (n=33)
PN (n=57)
Normal (n=11)
f
edge-like
core-like
astrocyte
HDAC10
HDAC2
HDAC1
HDAC3
HDAC8
HDAC7
HDAC11
HDAC9
HDAC5
HDAC4
HDAC6
g
edge-like
core-like
157
711
1079
1051
374
267
336
339
1005
HDAC1
β-actin
MW (kDa)
h
Olig2
HDAC1
Merge
CD109

Supplementary Figure 5 HDAC1 plays a role in the protumorigenic effect of core cells and is associated with poorer clinical outcome. **a** Representative bioluminescence images of mice injected with edge-like 1051 or core-like 267 and treated with vehicle or AR42. **b** mRNA expression of C/EBPβ in 59 matched longitudinal GBM samples (primary and recurrent tumors) grouped according to the expression of CD109 (up n = 30 patients or down n = 29 patients). **$p<0.01$ using unpaired, two-tailed t test. Data are mean ± s.d. **c** mRNA expression of HDAC1 in the same samples as in "b" grouped according to the tumor subtype (MES n = 26, PN n =15 and CL n = 18 independent samples). **$p<0.01$ using unpaired, two-tailed t test. Data are mean ± s.d.. **d** Analysis of IHC staining intensity (H-score) for HDAC1 in recurrent GBM tissues (left) and representative image of IHC staining (right). MES n = 5 independent samples; non-MES n = 8 independent samples; The line is the median. Scale bar 200 µm. **e** Analysis of mRNA expression of HDAC1 and HDAC2 in glioma tumor and normal brain samples from TCGA datasets, stratified according to molecular subtypes (MES n = 58, PN n = 57, Neural = 33, CL n = 54 and Normal n = 11 independent samples). ***$p<0.001$ using one-way ANOVA followed by Tukey's posttest. Data are mean ± s.d. **f** Heatmap generated from microarray data (GSE67089) showing expression of HDACs in edge-like and core-like GBM spheres, astrocytes and neural progenitor cells. **g** WB for HDAC1 using edge-like 157, 711, 1079, 1051, 374 and core-like 267, 336, 339, 1005 GBM spheres. **h** Representative immunofluorescent staining of human GBM tissues for HDAC1 (green), Olig2 (red) and DNA (blue) (upper) or for HDAC1 (green), CD109 (red) and DNA (blue) (lower). Scale bar 50 µm.

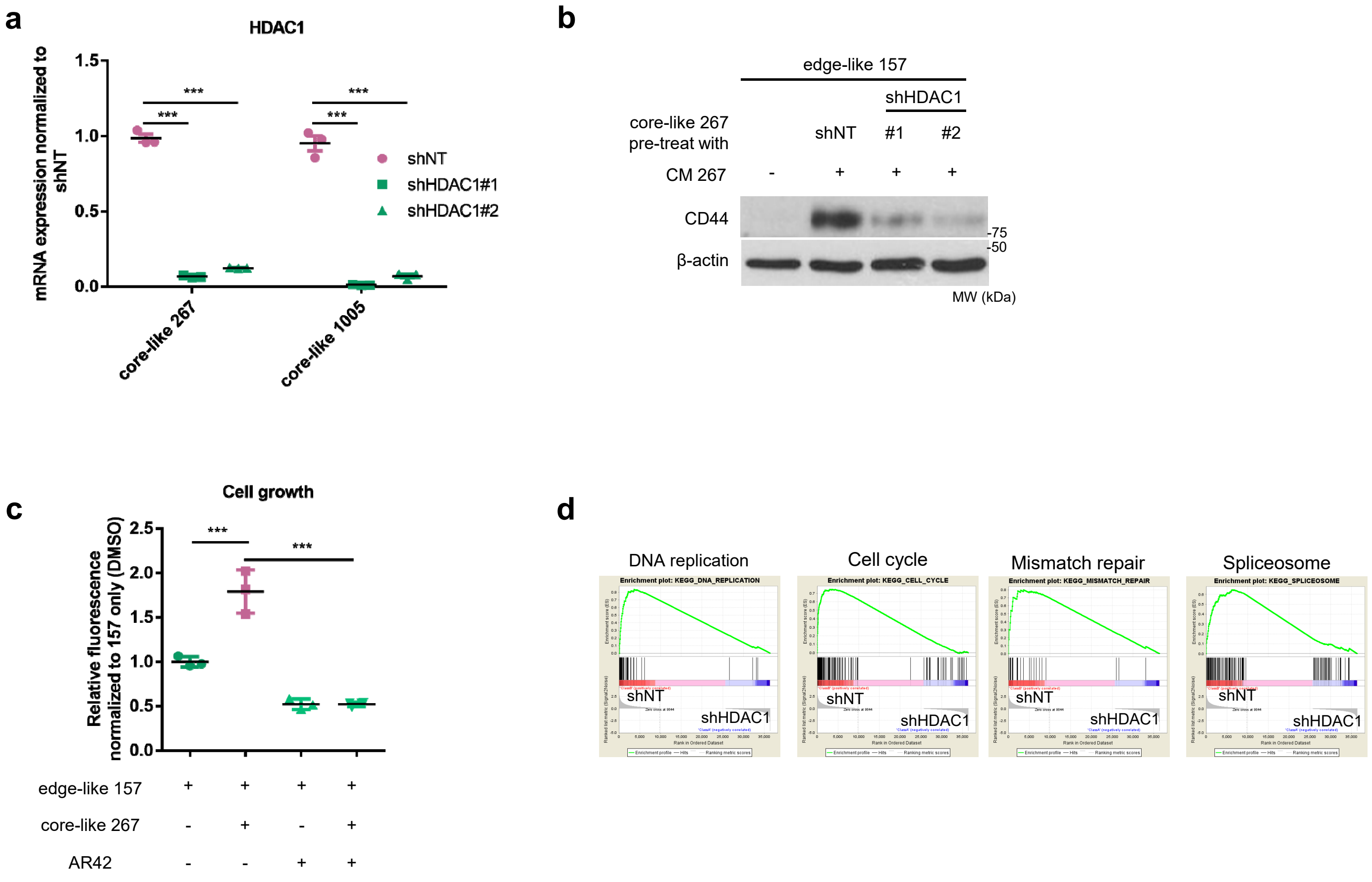
a
HDAC1
mRNA expression normalized to shNT
1.5
1.0
0.5
0.0
***
***
***
***
shNT
shHDAC1#1
shHDAC1#2
core-like 267
core-like 1005
b
edge-like 157
shHDAC1
core-like 267
pre-treat with
shNT
#1
#2
CM 267
-
+
+
+
CD44
β-actin
-75
-50
MW (kDa)
c
Cell growth
Relative fluorescence normalized to 157 only (DMSO)
2.5
2.0
1.5
1.0
0.5
0.0
***
***
edge-like 157 + + + +
core-like 267 - + - +
AR42 - - + +
d
DNA replication
Cell cycle
Mismatch repair
Spliceosome
shNT
shHDAC1
shNT
shHDAC1
shNT
shHDAC1
shNT
shHDAC1

Supplementary Figure 6 Inhibition of HDAC1 attenuates the intercellular signaling from core to edge GBM cells. **a** qRT-PCR analysis for HDAC1 using core-like 267 and 1005 GBM spheres infected with lentiviruses encoding shNT or shHDAC1. ***$p<0.001$ using one-way ANOVA followed by Dunnett's posttest. Data are mean ± s.d.; n = 3 independent samples per group. **b** WB for HDAC1 using core-like 267 GBM spheres infected with lentiviruses encoding shNT or shHDAC1. **c** Fluorescence intensity of mCherry of labeled edge-like 157 GBM spheres treated with AR42 or DMSO in the presence of unlabeled edge-like 157 or core-like 267 GBM spheres. ***$p<0.001$ using one-way ANOVA followed by Dunnett's posttest. Data are mean ± s.d.; n = 3 independent samples per group. **d** GSEA of shNT infected core-like 267 or 28 GBM spheres, as compared to shHDAC1 infected spheres. Gene sets shown are DNA repair, cell cycle and spliceosome-associated genes.

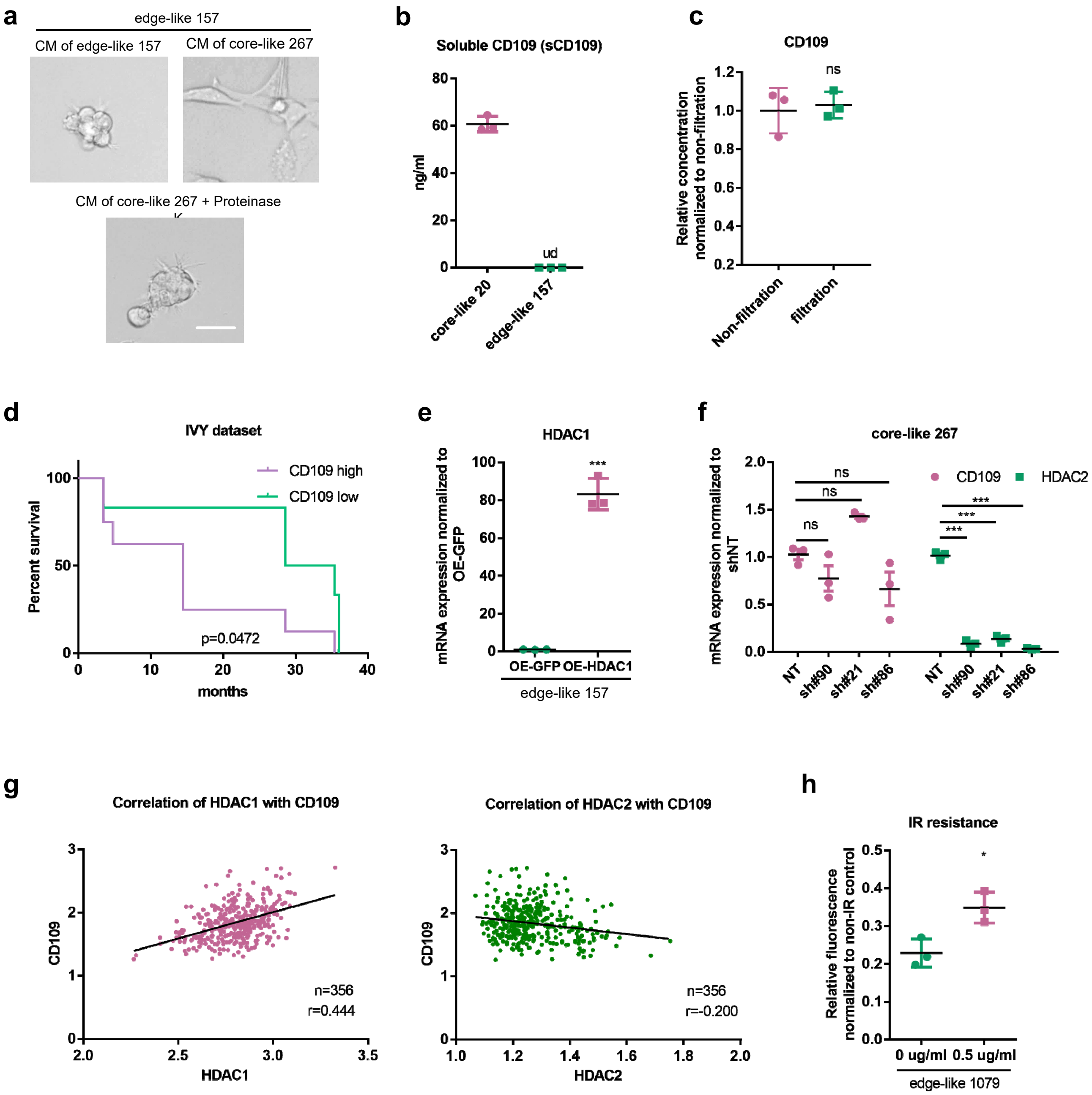
a
edge-like 157
CM of edge-like 157
CM of core-like 267
CM of core-like 267 + Proteinase K
b
Soluble CD109 (sCD109)
ng/ml
ud
core-like 20
edge-like 157
c
CD109
ns
Relative concentration normalized to non-filtration
Non-filtration
filtration
d
IVY dataset
CD109 high
CD109 low
Percent survival
p=0.0472
months
e
HDAC1
***
mRNA expression normalized to OE-GFP
OE-GFP
OE-HDAC1
edge-like 157
f
core-like 267
CD109
HDAC2
mRNA expression normalized to shNT
ns
***
NT
sh#90
sh#21
sh#86
g
Correlation of HDAC1 with CD109
CD109
HDAC1
n=356
r=0.444
Correlation of HDAC2 with CD109
HDAC2
n=356
r=-0.200
h
IR resistance
Relative fluorescence normalized to non-IR control
*
0 ug/ml
0.5 ug/ml
edge-like 1079

Supplementary Figure 7 Soluble CD109 is a mediator of HDAC1-derived intercellular signals from core to edge GBM cells. **a** Representative images of edge-like 157 GBM spheres treated with CM from edge-like 157 or core-like 267 GBM spheres pre-treated with proteinase K. Scale bars are 50 um. **b** Enzyme-linked immunosorbent assay (ELISA) of soluble CD109 in CM from edge-like 157 or core-like 20 GBM spheres. Data are mean ± s.d.; n = 3 independent samples per group, ud – undetected. **c** ELISA of secreted CD109 in CM from core-like 267 GBM spheres with/without filtration. Data are mean ± s.d.; n = 3 independent samples per group, ns - not significant, using unpaired, two-tailed t test. **d** Kaplan-Meier survival curve of GBM patients in IVY dataset with high or low CD109 expression in the leading edge. $p<0.05$ using two sided log-rank test; n = 6 animals per group. **e** qRT-PCR analysis of HDAC1 expression in edge-like 157 GBM spheres infected with lentiviruses encoding GFP or HDAC1. ***$p<0.001$, using unpaired, two-tailed t test. Data are mean ± s.d.; n = 3 independent samples per group. **f** qRT-PCR analysis of HDAC2 and CD109 expression in core-like 267 GBM spheres that were infected with shNT or shHDAC2 lentiviruses. ***$p<0.001$ using one-way ANOVA followed by Dunnett's posttest. Data are mean ± s.d.; n = 3 independent samples per group. **g** Correlation of HDAC1 and HDAC2 with CD109 expression levels in TCGA dataset. Pearson r = 0.444 and r = - 0.200 respectively. **h** *In vitro* cell viability assay of edge-like 1079 GBM spheres pretreated with recombinant CD109 and subsequently irradiated with 2Gy (IR). *$p<0.05$, using unpaired, two-tailed t test. Data are mean ± s.d.; n = 3 independent samples per group.

a

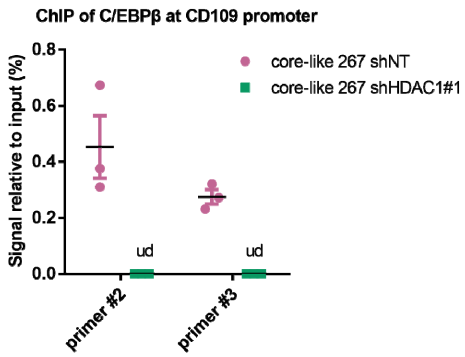
ChIP of C/EBPβ at CD109 promoter
Signal relative to input (%)
0.8
0.6
0.4
0.2
0.0
core-like 267 shNT
core-like 267 shHDAC1#1
ud
ud
primer #2
primer #3

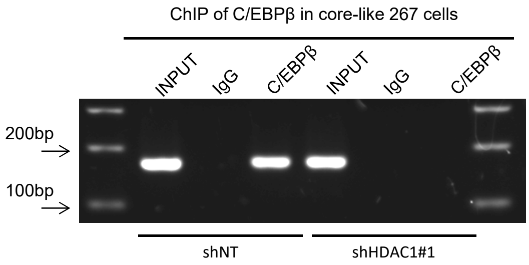
ChIP of C/EBPβ in core-like 267 cells
INPUT
IgG
C/EBPβ
INPUT
IgG
C/EBPβ
200bp
100bp
shNT
shHDAC1#1

b

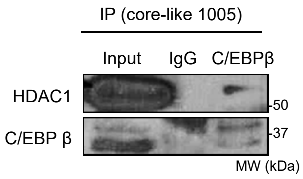
IP (core-like 1005)
Input
IgG
C/EBPβ
HDAC1
C/EBP β
50
37
MW (kDa)

Supplementary Figure 8 HDAC1 regulates transcription of CD109 via C/EBPβ. **a** ChIP analysis showing enrichment of C/EBPβ at CD109 promoter region in core-like 267 GBM spheres that were infected with shNT or shHDAC1 lentiviruses (left). ud - undetected. Data are mean ± s.d.; n = 3 independent samples per group. RT-PCR analysis of corresponding samples (right). **b** Co-immunoprecipitation of HDAC1 in core-like 1005 GBM spheres with antibodies against C/EBPβ.

a

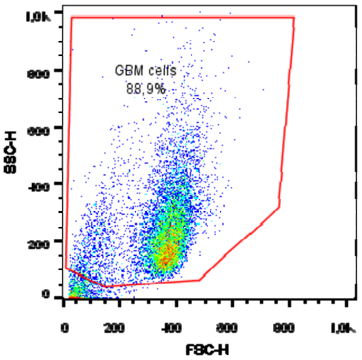
GBM cells
88.9%
SSC-H
FSC-H
0
200
400
600
800
1,0K

b

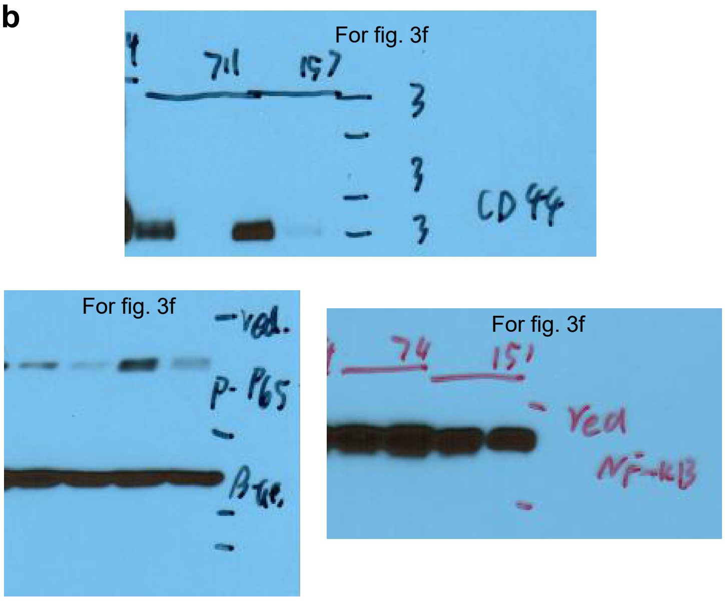
For fig. 3f
711
157
CD44
For fig. 3f
p-P65
For fig. 3f
74
151
NF-kB

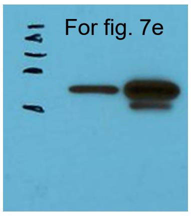
For fig. 7e

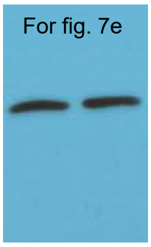
For fig. 7e

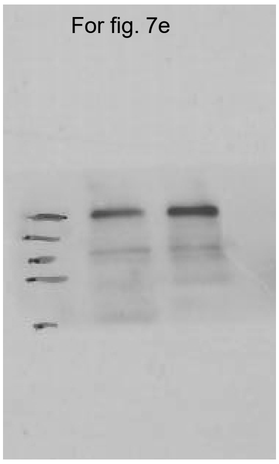
For fig. 7e

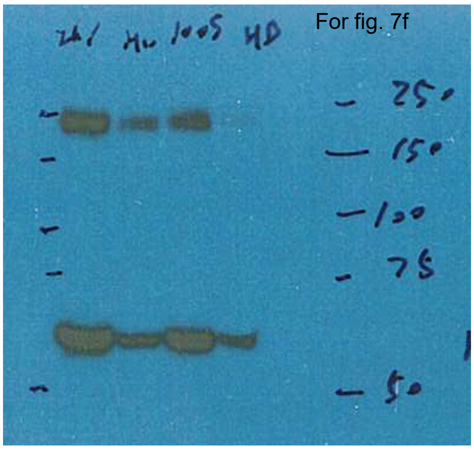
For fig. 7f
HD
250
150
100
75
50

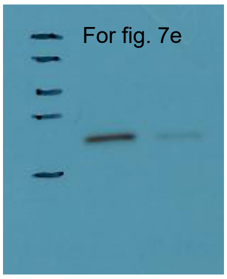
For fig. 7e

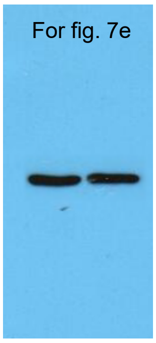
For fig. 7e

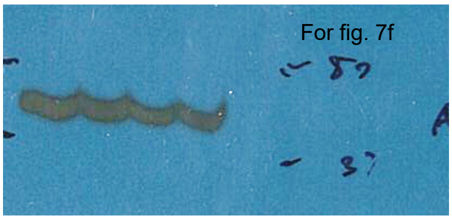
For fig. 7f
50
37

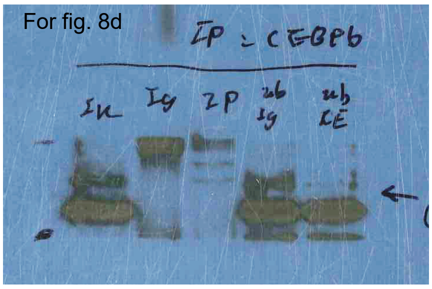
For fig. 8d
IP
In
Ig
IP

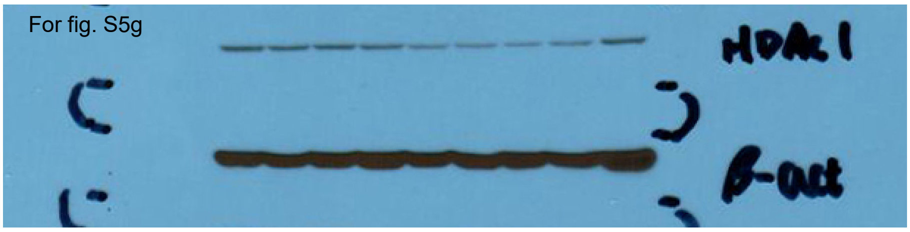
For fig. S5g
HDAC1

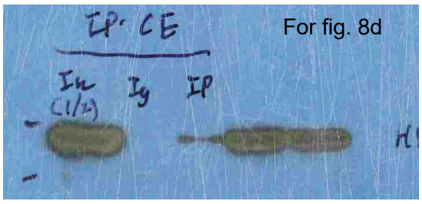
For fig. 8d
IP CE
In
(1/2)
Ig
IP

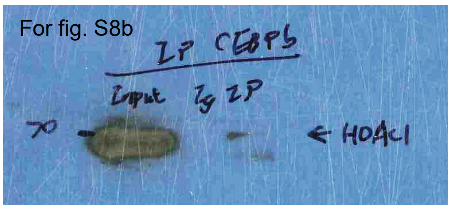
For fig. S8b
Input
Ig
IP
70
HDAC1

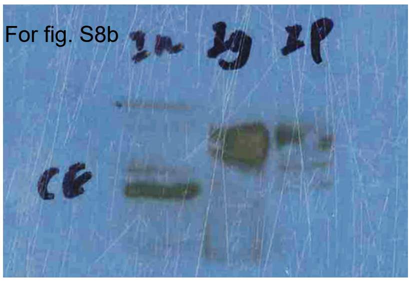
For fig. S8b
In
Ig
IP
CE

Supplementary Figure 9 Unprocessed data. **a** Flow cytometry gating used for apoptosis assay with CellEvent Caspase-3/7 Green Flow Cytometry Assay Kit. **b** Full scans of western blots presented in the paper.
