# Supplementary material for: Glioma-initiating cells at tumor edge gain signals from tumor core cells to promote their malignancy
Source: Nat Commun. 2020 Sep 16;11:4660. doi: 10.1038/s41467-020-18189-y (PMC7494913; doi:10.1038/s41467-020-18189-y)
Supplement: Supplementary file 3 — Description of Additional Supplementary Information [file 41467_2020_18189_MOESM3_ESM.pdf]

Description of Additional Supplementary Files

File Name: Supplementary Data 1
Description: List of all GBM sphere lines and the experiments in which the indicated spheres were used in the study. Expression of key edge/core markers is indicated.

File Name: Supplementary Data 2
Description: Comparison of the overall survival (OS) and progression free survival (PFS) of GBM patients that underwent complete or non-complete resection. Data were obtained from 15 previous studies.

File Name: Supplementary Data 3
Description: Results of LC-MS/MS protein identification in conditioned medium from core-like 267 and edge-like 157 GBM spheres.

File Name: Supplementary Data 4
Description: Results of LC-MS/MS protein identification in conditioned medium from 267 core-like GBM cells infected with shNT or shHDAC1 encoding lentiviruses.

File Name: Supplementary Data 5
Description: List of oligonucleotides and their sequences used in this study.

File Name: Supplementary Data 6
Description: Results of statistical analysis performed in this study.

File Name: Supplementary Movie 1
Description: 3D reconstruction of confocal microscopy images of mouse brain slice culture seeded with core 1051 sphere cells (green) and stained for Collagen IV (red) to label blood vessels (red).

File Name: Supplementary Movie 2
Description: 3D reconstruction of confocal microscopy images of mouse brain slice culture seeded with edge 1051 sphere cells (blue) and stained for Collagen IV (red) to label blood vessels (red).
