# Supplementary material for: Glioma-initiating cells at tumor edge gain signals from tumor core cells to promote their malignancy
Source: Nat Commun. 2020 Sep 16;11:4660. doi: 10.1038/s41467-020-18189-y (PMC7494913; doi:10.1038/s41467-020-18189-y)
Supplement: Supplementary file 12 — Reporting summary [file 41467_2020_18189_MOESM12_ESM.pdf]

naturereserach

Corresponding author(s): Ichiro Nakano

Last updated by author(s): Jun 25, 2020

Reporting Summary

Nature Research wishes to improve the reproducibility of the work that we publish. This form provides structure for consistency and transparency in reporting. For further information on Nature Research policies, seeAuthors & Referees and theEditorial Policy Checklist .

Statistics

For all statistical analyses, confirm that the following items are present in the figure legend, table legend, main text, or Methods section.

n/a	Confirmed	
☐	☒	The exact sample size (*n*) for each experimental group/condition, given as a discrete number and unit of measurement
☐	☒	A statement on whether measurements were taken from distinct samples or whether the same sample was measured repeatedly
☐	☒	The statistical test(s) used AND whether they are one- or two-sided<br>*Only common tests should be described solely by name; describe more complex techniques in the Methods section.*
☒	☐	A description of all covariates tested
☒	☐	A description of any assumptions or corrections, such as tests of normality and adjustment for multiple comparisons
☐	☒	A full description of the statistical parameters including central tendency (e.g. means) or other basic estimates (e.g. regression coefficient) AND variation (e.g. standard deviation) or associated estimates of uncertainty (e.g. confidence intervals)
☐	☒	For null hypothesis testing, the test statistic (e.g. *F*, *t*, *r*) with confidence intervals, effect sizes, degrees of freedom and *P* value noted<br>*Give P values as exact values whenever suitable.*
☒	☐	For Bayesian analysis, information on the choice of priors and Markov chain Monte Carlo settings
☒	☐	For hierarchical and complex designs, identification of the appropriate level for tests and full reporting of outcomes
☐	☒	Estimates of effect sizes (e.g. Cohen's *d*, Pearson's *r*), indicating how they were calculated

Our web collection on statistics for biologists contains articles on many of the points above.

Software and code

Policy information about availability of computer code

Data collection	NIS-Elements version 4.00 (for confocal microscopy); Attune NxT version 4.2 (for flow cytometry)
Data analysis	GraphPad software version 6.0 (GraphPad Software); IBM SPSS Statistics version 22.0 (SPSS); Gene Cluster version 3.0, Gene Tree View version 3.0; FlowJo version 10; STAR version 2.5.3a; Cufflinks version 2.2.1;

For manuscripts utilizing custom algorithms or software that are central to the research but not yet described in published literature, software must be made available to editors/reviewers. We strongly encourage code deposition in a community repository (e.g. GitHub). See the Nature Research guidelines for submitting code & software for further information.

Data

Policy information about availability of data

All manuscripts must include a data availability statement. This statement should provide the following information, where applicable:

- Accession codes, unique identifiers, or web links for publicly available datasets
- A list of figures that have associated raw data
- A description of any restrictions on data availability

RNAseq data of 12 GBM sphere lines as well as all RNAseq data from Fig.1 (9 tissue samples) have been deposited at the Gene Expression Omnibus database under the accession code GSE153746. All the other data supporting the findings of this study are available within the article and its supplementary information files.
Databases:
The Ivy Glioblastoma Atlas Project (https://glioblastoma.alleninstitute.org/)
GBMseq (http://www.gbmseq.org/)
Repository for Molecular Brain Neoplasia Data (https://caintergator.nci.nih.gov/rembrandt)

Field-specific reporting

Please select the one below that is the best fit for your research. If you are not sure, read the appropriate sections before making your selection.

☒ Life sciences ☐ Behavioural & social sciences ☐ Ecological, evolutionary & environmental sciences

For a reference copy of the document with all sections, see nature.com/documents/nr-reporting-summary-flat.pdf

Life sciences study design

All studies must disclose on these points even when the disclosure is negative.

Sample size	Sample size was determined on the basis of our previous studies (Clin Cancer Res. 2013, 19(3): 631–642; J Clin Invest. 2017, 127(8): 3075-3089; Cancer Cell. 2018, 34(1): 119-135) and the experience of the authors. We expect changes in the gene/protein expression and function measurements to be detected with n=3 samples per group.
Data exclusions	No data were excluded from the analyses. All cells/animals that met proper experimental conditions were included in the analysis.
Replication	All experimental findings were repeated using multiple patient-derived glioma sphere models with at least 3 biological replicates in each experiment. Precise numbers of biological replicates are given in the figure legends. All experimental findings were reliably reproduced.
Randomization	Since this is not a clinical study, no randomization was necessary.
Blinding	Since this is not a clinical study, no blinding was necessary.

Reporting for specific materials, systems and methods

We require information from authors about some types of materials, experimental systems and methods used in many studies. Here, indicate whether each material, system or method listed is relevant to your study. If you are not sure if a list item applies to your research, read the appropriate section before selecting a response.

Materials & experimental systems

n/a	Involved in the study	
☐	☒	Antibodies
☐	☒	Eukaryotic cell lines
☒	☐	Palaeontology
☐	☒	Animals and other organisms
☐	☒	Human research participants
☒	☐	Clinical data

Methods

n/a	Involved in the study	
☒	☐	ChIP-seq
☐	☒	Flow cytometry
☒	☐	MRI-based neuroimaging

Antibodies

Antibodies used

The following antibodies were used in the study (Epitope, Supplier, catalogue number, experiment type, host)
anti-CD44 (Cell Signaling, 3570; IHC, WB, IF; mouse),
anti-GAPDH (Abcam, ab9485 ; WB; rabbit),
anti-β-Actin (Cell Signaling, 3700; WB),
anti-CD109 (Santa Cruz sc-271085; WB; mouse),
anti-C/EBPβ (Genetex GTX100675; ChIP, rabbit; Novus NBP2-67635; IP; rabbit),
anti-N-cadherin (BD Biosciences 610920; IF; mouse),
anti-Olig2 (Millipore AB9610; IHC, IF; rabbit),
anti-mCherry (Novus NBP2-25157ss; IHC; rabbit),
anti-GFP (Abcam ab6673; IF; goat),
anti-p-p65 (Cell Signaling 3033s; WB; rabbit),
anti-p65 (Cell Signaling 8242s; WB; rabbit),
anti-HDAC1 (Cell Signaling 34589s; WB, ChIP; rabbit; 5356s; WB; mouse),
anti-c-Myc (Santa cruz sc-40, IHC,IF; mouse),
anti-KRAS (ThermoFisher, 415700; IHC; mouse),
anti-CHEK1 (Abcam 40866; IHC; rabbit),
anti-rabbit IgG-Alexa Fluor 555 (Cell Signaling, 4413; IF)
anti-mouse IgG-Alexa Fluor 647 (Cell Signaling, 4410; IF)
anti-mouse IgG-Horseradish peroxidase (GE Healthcare, NXA931; WB)

	anti-rabbit IgG-Alexa Fluor 488 (Cell Signaling, 4412; IF)
Validation	All antibodies were used as per manufacturer's recommendations. Each primary antibody data provided in the manuscript has been validated for the species and application on the manufacturer's website

Eukaryotic cell lines

Policy information about cell lines

Cell line source(s)	All glioma sphere models were derived from disassociation of patient specimens. Clinical glioma specimens samples were collected in the Department of Pathology and Laboratory Medicine at UAB (sample 1005, 1014, 1020, 1027, 1037, 1051, 1079, 0573), UCLA (sample 157, 339, 374, 408), MDA (sample 267, 20, 28, 711). 293FT cells were purchased from Thermo Fisher Scientific.
Authentication	All glioma sphere cell lines were verified for purity using STR analysis. 293FT cells were supplied by Thermo Fisher Scientific and therefore authentication was not performed.
Mycoplasma contamination	All cell lines were regularly tested for mycoplasma contamination. All cells were free from mycoplasma at the time of all experiments.
Commonly misidentified lines (See ICLAC register)	None of the cell lines used are listed in the ICLAC list.

Animals and other organisms

Policy information about studies involving animals; ARRIVE guidelines recommended for reporting animal research

Laboratory animals	6-8 weeks old female NOD SCID mice were used in this study. The mice were housed in groups of five animals per cage and had access to autoclaved water and pelleted feed. The cage environment was enriched with a mouse house. The mice were kept at a standard temperature of 22 C±2 C and a relative humidity of 55% (45-70%) in a 12:12-hour light:dark cycle (lights on, 6 am to 6 pm).
Wild animals	The study did not involve wild animals.
Field-collected samples	The study did not involve samples collected from the field.
Ethics oversight	This study was conducted under the approved Institutional Review Board (IRB) and Institutional Animal Care and Use Committee (IACUC) protocols in University of Alabama at Birmingham (UAB), University of California Los Angeles (UCLA) and MD Anderson Cancer Center (MDA). The IRB Protocol number at UAB is #N151013001.

Note that full information on the approval of the study protocol must also be provided in the manuscript.

Human research participants

Policy information about studies involving human research participants

Population characteristics	No population characteristics were applied to this study.
Recruitment	No recruitment was applied to this study.
Ethics oversight	All patients whose excess tissue specimens were analyzed in this study were informed and consented according to respective IRB protocols in University of Alabama at Birmingham (UAB), University of California Los Angeles (UCLA) and MD Anderson Cancer Center (MDA).

Note that full information on the approval of the study protocol must also be provided in the manuscript.

Flow Cytometry

Plots

Confirm that:

- [x] The axis labels state the marker and fluorochrome used (e.g. CD4-FITC).
- [x] The axis scales are clearly visible. Include numbers along axes only for bottom left plot of group (a 'group' is an analysis of identical markers).
- [x] All plots are contour plots with outliers or pseudocolor plots.
- [x] A numerical value for number of cells or percentage (with statistics) is provided.

Methodology

Sample preparation	Patient derived Edge or Core GBM cells were cultivated for 3 days in the presence or absence of rhCD109 and subsequently irradiated (8 Gy). 3 days later cells were stained with CellEvent Caspase-3/7 Green Flow Cytometry Assay Kit (Thermo scientific) according to the manufacturer's protocol.
Instrument	Attune NxT Flow Cytometer (Thermo scientific)
Software	FlowJo 10
Cell population abundance	All GBM cells obtained after the dissociation of neurospheres were analyzed without separation into different populations.
Gating strategy	The samples were gated by FSC-H and SSC-H to distinguish cells from debris.

☒ Tick this box to confirm that a figure exemplifying the gating strategy is provided in the Supplementary Information.
